# Co-expression of HLA-E and HLA-G on genetically modified porcine endothelial cells attenuates human NK cell-mediated degranulation

**DOI:** 10.3389/fimmu.2023.1217809

**Published:** 2023-07-17

**Authors:** Arthur A. Cross-Najafi, Kristine Farag, Abdulkadir Isidan, Wei Li, Wenjun Zhang, Zhansong Lin, Julia R. Walsh, Kevin Lopez, Yujin Park, Nancy G. Higgins, David K.C. Cooper, Burcin Ekser, Ping Li

**Affiliations:** ^1^ Transplant Division, Department of Surgery, Indiana University School of Medicine, Indianapolis, IN, United States; ^2^ Department of Microbiology and Immunology, Indiana University School of Medicine, Indianapolis, IN, United States; ^3^ Ragon Institute of Massachusetts General Hospital (MGH), Massachusetts Institute of Technology (MIT), and Harvard, Cambridge, MA, United States; ^4^ Transplant Immunology, Indiana University Health, Indianapolis, IN, United States; ^5^ Center for Transplantation Sciences, Massachusetts General Hospital/Harvard Medical School, Cambridge, MA, United States

**Keywords:** xenotransplantation, natural killer cells, immune tolerance, immune rejection, HLA-E, HLA-G, inhibitory ligands and receptors, degranulation

## Abstract

Natural killer (NK) cells play an important role in immune rejection in solid organ transplantation. To mitigate human NK cell activation in xenotransplantation, introducing inhibitory ligands on xenografts *via* genetic engineering of pigs may protect the graft from human NK cell-mediated cytotoxicity and ultimately improve xenograft survival. In this study, non-classical HLA class I molecules HLA-E and HLA-G were introduced in an immortalized porcine liver endothelial cell line with disruption of five genes (*GGTA1*, *CMAH*, *β4galNT2*, *SLA-I α chain*, and *β-2 microglobulin*) encoding three major carbohydrate xenoantigens (αGal, Neu5Gc, and Sda) and swine leukocyte antigen class I (SLA-I) molecules. Expression of HLA-E and/or HLA-G on pig cells were confirmed by flow cytometry. Endogenous HLA-G molecules as well as exogenous HLA-G VL9 peptide could dramatically enhance HLA-E expression on transfected pig cells. We found that co-expression of HLA-E and HLA-G on porcine cells led to a significant reduction in human NK cell activation compared to the cells expressing HLA-E or HLA-G alone and the parental cell line. NK cell activation was assessed by analysis of CD107a expression in CD3^-^CD56^+^ population gated from human peripheral blood mononuclear cells. CD107a is a sensitive marker of NK cell activation and correlates with NK cell degranulation and cytotoxicity. HLA-E and/or HLA-G on pig cells did not show reactivity to human sera IgG and IgM antibodies. This *in vitro* study demonstrated that co-expression of HLA-E and HLA-G on genetically modified porcine endothelial cells provided a superior inhibition in human xenoreactive NK cells, which may guide further genetic engineering of pigs to prevent human NK cell mediated rejection.

## Introduction

Pig-to-human xenotransplantation offers a promising solution to address the persistent organ shortage (1). Interspecies incompatibilities result in robust human immune responses directed against the porcine xenograft. The consequence is rapid destruction and failure of the transplanted organ. Genetic modification (GM) of pigs has proven to be a valuable strategy for improving pig-human compatibility (2). Recent advancements in the genetic engineering of pigs have brought us closer to achieving successful xenotransplantation (3). In 2022, the first genetically modified pig-to-human cardiac xenotransplant was performed, which kept the recipient alive for two months (4). This groundbreaking event marks an important turning point: hyperacute xenograft rejection is no longer an absolute contraindication to xenotransplantation. Despite this exciting fact, acute and chronic organ rejection remain major barriers to successful pig-to-human xenotransplantation. To achieve long-term survival of pig xenografts and reduce the need for life-long immunosuppressive therapy with deleterious side effects, further GMs of pig tissues and organs are needed. These GMs will aim to reduce cell-mediated immune responses and improve major histocompatibility complex (MHC) compatibilities (5–10).

Human NK cells comprise the first line of defense of the innate immune system and are also involved in adaptive immunity. In solid organ transplantation, NK cell infiltration has been characterized with increased graft rejection in both allografts and xenografts (11, 12). NK cells can discriminate self, non-self, and abnormal cells (virus-infected cells or tumor cells) quickly, using a variety of cell-surface receptors which interact with the ligands on target cells (13). The balance of inhibitory and activating signals determines NK cell activation or inhibition. NK cell inhibitory ligands such as non-classical human leukocyte antigens (HLA)-E and -G are highly expressed in the human placenta (14), and contribute to establishing and maintaining immune tolerance at the maternal-fetal interface (15). Attempts have been made to investigate the role of HLA-E and HLA-G on porcine cells in regulating human NK cell activation *in vitro* and different inhibition pathways have been revealed (16, 17). Unlike classical HLA class I molecules, HLA-E and HLA-G display a limited polymorphism and are not considered in HLA typing for allotransplantation (18, 19). HLA-G plays an immunomodulatory role by binding the inhibitory receptors: Ig-like transcript 2 (ILT2) on dendritic cells, B cells, NK cells, and T cells; ILT4 on cells of myeloid origin; and killer cell immunoglobulin-like receptor 2DL4 (KIR2DL4) on NK cells (20–23). HLA-G expression is beneficial and promotes graft tolerance in solid organ transplantation, as evidenced by increased HLA-G expression in allografts and/or plasma correlating with improved graft acceptance (24, 25). Forte et al. reported that HLA-G expression inhibits the rolling adhesion of activated human NK cells on porcine endothelial cells (26) and partially protects porcine cells against direct human NK cytotoxicity (27). The protective role of HLA-E on porcine cells in human NK cell-mediated cytotoxicity has been reported (10, 28, 29). The HLA-E molecules present a highly conserved set of nonameric peptides (VL9) derived from the leader sequence of HLA-A/B/C/G molecules to NK cells and specific CD8 T cells (30). HLA-E-VL9 complex is a major inhibitory ligand for the NK inhibitory receptor NKG2A (31). VL9 peptides stabilize HLA-E molecules and determine HLA-E expression on cell surface. Previous studies demonstrated that HLA-E molecule loaded with the HLA-G leader peptide exhibited the highest affinity for NKG2A receptor (32) and co-expression HLA-G and HLA-E on swine endothelial cells efficiently enhanced the inhibition of NK cell-mediated cytotoxicity (33). Recent *ex vivo* studies indicated that transgenic expression of HLA-E attenuated porcine lung xenograft injury and reduced NK cell recruitment in pig limbs when perfused with human blood (34, 35).

In this study, an immortalized porcine liver-derived endothelial cell line (ipLDEC) with five-gene knockout (5GKO) (36) was used to express HLA-E, HLA-G, or co-express HLA-E and HLA-G molecules, namely 5GKO.HLA-E, 5GKO.HLA-G, and 5GKO.HLA-E.HLA-G cells. Human NK cell responses to these three modified cells as well as the parental 5GKO cells were evaluated by examining CD107a surface expression on CD3^-^CD56^+^ population. CD107a, also known as lysosomal-associated membrane protein-1 (LAMP-1), is a functional marker for NK cell activation, which correlates with both cytokine secretion and NK cell-mediated cytotoxicity (37). The reactivity of human antibodies to HLA-E and/or HLA-G-expressing porcine cells was examined by a flow cytometry-based assay.

## Materials and methods

### Establishment of genetically modified porcine endothelial cell lines

The five-gene knockout cell line (5GKO, *GGTA1*/*CMAH*/*β4galNT2*/*SLA-I α chain*/*β-2 microglobulin*) was generated from ipLDEC, as previously described (36). The 5GKO cell line served as the parental cell line to express HLA-E and/or HLA-G molecules.

**Figure 1:**
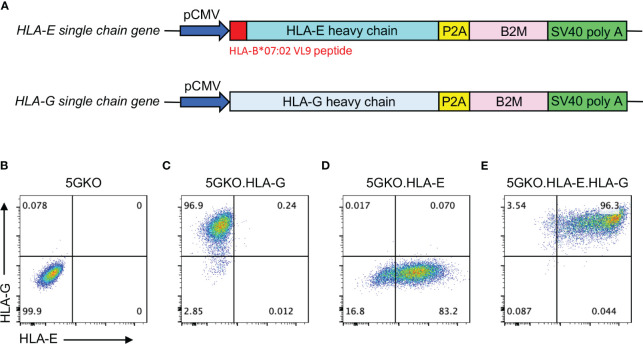
Expression of HLA-E and/or HLA-G on 5GKO cells. **(A)** Schematic of expression cassettes with a single chain gene of HLA-E and HLA-G. Flow cytometry analysis of cell surface expression of HLA-E and HLA-G on 5GKO **(B)**, 5GKO.HLA-G **(C)**, 5GKO.HLA-E **(D)**, and 5GKO.HLA-E.HLA-G **(E)** cell lines.

HLA-G is a heterodimer protein consisting of a heavy chain and β-2 microglobulin (B2M) subunits encoded by two genes located on different chromosomes. A single chain gene was designed by linking the *HLA-G heavy chain* (NCBI reference number: NM_001363567.2) and *B2M* (NCBI reference number: NM_004048.4) genes with self-cleaving peptide P2A DNA fragment, synthesized by Integrated DNA Technologies (IDT, Coralville, IA), and inserted downstream of the CMV promoter in an expression vector derived from pEGFP-N1, which had *EGFP* gene removed (**Figure 1A**). This recombinant plasmid was delivered into 5GKO ipLDEC by electroporation using the Neon Transfection System (Thermo Fisher Scientific, Waltham, MA). The transfected cells were cultured in selective media containing G418 at 200 ng/mL for 10 days. HLA-G expression was verified by flow cytometry using PE-conjugated mouse anti-HLA-G antibody (Clone 87G, BioLegend, San Diego, CA). 5GKO cells were used as a control (**Figure 1B**). 5GKO.HLA-G cells were isolated by a BD FACSAria Fusion cell sorter (BD Biosciences, San Jose, CA) (**Figure 1C**).

The HLA-E molecule is a trimeric complex, consisting of a heavy chain, B2M, and a signal peptide derived from other HLA class I molecules (30). To ensure HLA-E expression in porcine cells, the *HLA-E heavy chain* gene (HLA-E*010301 allele, NCBI reference number: NM_005516.6) was modified by replacing its original signal peptide DNA sequence with HLA-B*07:02 signal peptide (VMAPRTVLL, NCBI Reference Sequence: NM_005514.8) DNA sequence, then linked to *B2M* gene with P2A DNA fragment. This single-chain *HLA-E* gene was synthesized by IDT and subsequently cloned to the downstream of the CMV promoter in an expression vector derived from pEGFP-N1 (**Figure 1A**). This plasmid was delivered into 5GKO and 5GKO.HLA-G cells by electroporation, respectively. The transfected cells were cultured in selective media containing G418 at 200 ng/mL for 10 days. HLA-E expression was confirmed by flow cytometry using APC-conjugated mouse anti-HLA-E antibody (Clone 3D12, BioLegend). 5GKO.HLA-E and 5GKO.HLA-E.HLA-G cells were isolated by a BD FACSAria Fusion cell sorter (BD Biosciences) using APC-conjugated mouse anti-HLA-E antibody and PE-conjugated mouse anti-HLA-G antibody (BioLegend) (**Figures 1D, E**). Both HLA-E antibody and HLA-G antibody are specific. Cross-reactivity of HLA-E antibody to HLA-G molecules or HLA-G antibody to HLA-E molecules has not been observed.

### Stability and expression level of HLA-E and HLA-G on 5GKO cell lines

HLA-E and HLA-G surface expression on 5GKO cells were examined four times for three weeks after flow sorting. 5GKO.HLA-E, 5GKO.HLA-G, and 5GKO.HLA-E.HLA-G were stained with APC-conjugated mouse anti-HLA-E antibody and PE-conjugated mouse anti-HLA-G antibody (BioLegend), as described above. HLA-E and HLA-G expression were measured by the percentage of positive cells as well as the mean fluorescence of intensity (MFI). The percentage of HLA-E or HLA-G positive cells was compared between 5GKO.HLA-E and 5GKO.HLA-E.HLA-G cells or between 5GKO.HLA-G and 5GKO.HLA-E.HLA-G cells at each time point. HLA-E MFI was compared between 5GKO.HLA-E and 5GKO.HLA-E.HLA-G cells while HLA-G MFI was compared between 5GKO.HLA-G and 5GKO.HLA-E.HLA-G cells.

### HLA-E expression on 5GKO.HLA-E cells pulsed with HLA-G VL9 peptides

HLA-G VL9 peptide (VMAPRTLFL) was synthesized at purity of 95.1% by GenScript Biotech (Piscataway, NJ). 5GKO.HLA-E cells were incubated with HLA-G peptides at final concentrations of 25 µM, 50 µM, and 100 µM in a CO_2_ incubator at 37°C overnight. 5GKO.HLA-E cells alone were used as a control. HLA-E expression on pig cells was measured by APC-conjugated mouse anti-HLA-E antibody staining and analyzed by an LSRFortessa flow cytometer (BD Biosciences). Experiments were repeated three times with similar results.

### Human NK cell degranulation in response to porcine endothelial cell stimulation

NK cell degranulation assay was performed in a similar fashion as previously described (36, 38). CD107a is a functional marker and widely used for identification of NK cell activity (37, 39, 40). Commercially available buffy coats were acquired from Versiti Indiana Blood Center. Fresh whole blood was drawn from two human donors following the guidelines of the Institutional Review Board (IRB) of Indiana University, IRB#11013. Human peripheral blood mononuclear cells (PBMCs) were isolated from buffy coat and fresh whole blood using Ficoll-Paque Plus (GE-Healthcare, Pittsburgh, PA) and Lymphoprep (STEMCELL Technologies, Vancouver, Canada) gradient centrifugation, respectively, for a total of 5 human donors. PBMCs from 5 donors were cultured in RPMI1640 with 10% FBS, 1% penicillin/streptomycin, and 20 ng/mL recombinant human IL-2 (rhIL-2) (BioLegend) at 37°C in a 5% CO_2_ incubator for 5 days. 5GKO, 5GKO.HLA-E, 5GKO.HLA-G, and 5GKO.HLA-E.HLA-G cells were plated at 5 ×10^4^ per well in a Biocoat collagen I-coated 48-well plate (Corning Incorporated, Corning, NY) one day prior to co-culture. PBMCs were added to porcine cells at 5 ×10^5^ per well and co-cultured for 2 hours at 37°C in a CO_2_ incubator. Cultured cells were then collected and stained with fixable viability dye eFluor 780 (Thermo Fisher Scientific) and fluorochrome-conjugated antibodies against human CD45, CD3, CD56, and CD107a (BioLegend). Cells were fixed with 2% PFA for 15 minutes at room temperature and subsequently analyzed using an LSRFortessa flow cytometer (BD Biosciences). 70,000 - 80,000 events were acquired in lymphocyte gate. After pre-gating on CD45^+^ live singlets, NK cell degranulation activity was assessed by the percentage of CD107a positive cells in a CD3^-^CD56^+^ cell population. Flow data were analyzed using FlowJo v10 software (BD Biosciences). The experiment was repeated three times to obtain technical replicates.

### Human serum antibody reactivity to porcine endothelial cells expressing HLA-E and HLA-G

Human antibody binding to porcine endothelial cells was examined, as previously described (36). Briefly, 2×10^5^ porcine cells (5GKO, 5GKO.HLA-E, 5GKO.HLA-G, and 5GKO.HLA-E.HLA-G) were washed and incubated with 25% heat-inactivated human serum in EX-CELL 610-HSF serum-free medium (Sigma, St. Louis, MO) with 0.1% sodium azide for 1 hour at 4°C. Human sera were obtained from patients on the kidney transplant waitlist, 10 sera with high panel reactive antibody (PRA) and 10 sera with low PRA, for a total of 20 samples (n = 20). Each pig cell line was washed three times with EX-CELL 610-HSF serum-free medium and stained with goat anti-human IgG Alexa Fluor 488 or donkey anti-human IgM Alexa Fluor 647 (Jackson ImmunoResearch Laboratories Inc., West Grove, PA) for 30 minutes at 4°C, respectively. Cells were washed, fixed with 2% PFA for 15 minutes at room temperature, and subsequently analyzed using an LSRFortessa flow cytometer (BD Biosciences). Flow data were analyzed using FlowJo v10 software (BD Biosciences). Each pig cell line stained with goat anti-human IgG Alexa Fluor 488 or donkey anti-human IgM Alexa Fluor 647 was used as background and subtracted from each corresponding sera binding group. Human IgG and IgM bindings to pig cells were analyzed by stratification into low PRA and high PRA sera groups. The difference between low PRA and high PRA sera binding to each individual modified cell line was also compared.

### Statistical analysis

Statistical analyses were performed using GraphPad Prism 9 software (GraphPad Software, San Diego, CA). A normality test was used to assess data distribution. An ordinary one-way ANOVA multiple comparisons test with Šídák’s correction was used to analyze the differences among multiple groups. Student’s t-test was used to analyze the differences between the two groups. A p-value less than 0.05 was considered statistically significant.

## Results

### Expression of HLA-E and/or HLA-G molecules on porcine endothelial cells

**Figure 2:**
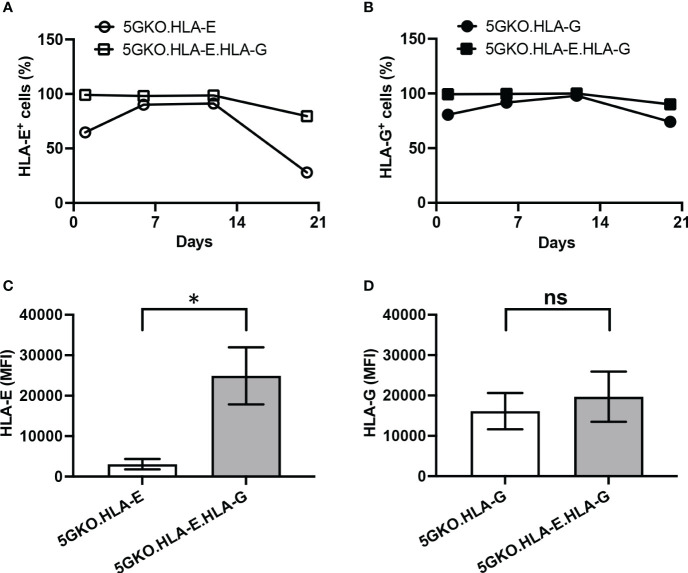
Comparison of HLA-E and HLA-G stability and cell surface expression on porcine cells. The percentage of HLA-E positive cells **(A)** and HLA-G positive cells **(B)** in modified pig cells were examined at different time points. **(C)** Abundance of cell surface HLA-E was compared between 5GKO.HLA-E and 5GKO.HLA-E.HLA-G cell lines. **(D)** Abundance of cell surface HLA-G was compared between 5GKO.HLA-G and 5GKO.HLA-E.HLA-G cell lines. Data presented as mean ± SEM. Student’s t-test was used to analyze the difference between two groups. ns, not significant; *p < 0.05.

Three porcine cell lines expressing HLA-E, HLA-G, and co-expressing HLA-E and HLA-G were successfully established (**Figures 1C–E**). Stability of HLA-E and HLA-G molecules on porcine cells was examined. During the three-week culture, HLA-E expression on 5GKO.HLA-E.HLA-G was much more stable compared to HLA-E on 5GKO.HLA-E cells as indicated by the percentage of HLA-E positive cells (**Figure 2A**). In addition, 5GKO.HLA-E.HLA-G cells exhibited significantly higher HLA-E expression than 5GKO.HLA-E cells (p < 0.05) (**Figure 2C**). HLA-G expression was comparable between 5GKO.HLA-G and 5GKO.HLA-E.HLA-G cells (**Figures 2B, D**). These results indicate that HLA-E molecules are stable and more highly expressed on 5GKO.HLA-E.HLA-G cells than on 5GKO.HLA-E cells. All cells were examined by flow cytometry to ensure HLA-E or HLA-G expression prior to being used in the functional assays.

### Enhanced HLA-E expression on 5GKO.HLA-E cells by pulsing exogenous HLA-G VL9 peptides

**Figure 3:**
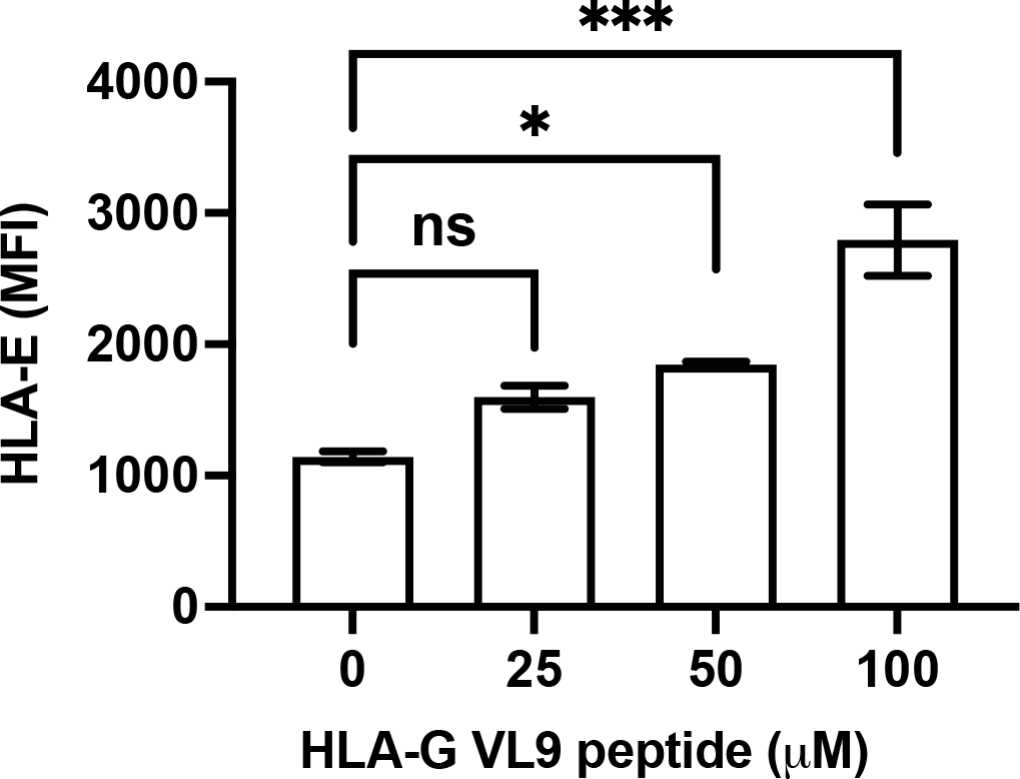
Increased HLA-E expression on 5GKO.HLA-E cells after pulsing with HLA-G VL9 peptides. 5GKO.HLA-E cells were incubated with HLA-G VL9 peptides at 25 µM, 50 µM, or 100 µM overnight. HLA-E expression was analyzed by flow cytometry using APC-conjugated mouse anti-HLA-E antibody. ns, not significant; *p < 0.05; ***p<0.001.

A recent study indicated that HLA class I signal peptide polymorphism influences surface HLA-E expression as well as NKG2A-HLA-E engagement (41). Surface HLA-E is unstable and is rapidly internalized (42). In 5GKO.HLA-E.HLA-G cells, HLA-E can bind to either HLA-B*07:02 VL9 or HLA-G VL9 peptides. In 5GKO.HLA-E cells, HLA-E can only bind HLA-B*07:02 VL9 peptides. To understand the mechanism by which 5GKO.HLA-E.HLA-G cells exhibited much higher HLA-E expression than 5GKO.HLA-E cells, we determined whether HLA-G VL9 peptide could enhance HLA-E expression on 5GKO.HLA-E cells. 5GKO.HLA-E cells were pulsed with HLA-G VL9 peptides at 25 µM, 50 µM, or 100 µM, and incubated overnight. HLA-E surface expression by flow cytometric analysis revealed that exogenous HLA-G VL9 peptides could significantly increase HLA-E expression on 5GKO.HLA-E cells in a dose dependent manner (**Figure 3**). This result suggests that HLA-G VL9 peptides can stabilize HLA-E molecules and enhance HLA-E expression on 5GKO.HLA-E cells.

### Inhibition of human NK cell degranulation by co-expression of HLA-E and HLA-G on porcine endothelial cells

**Figure 4:**
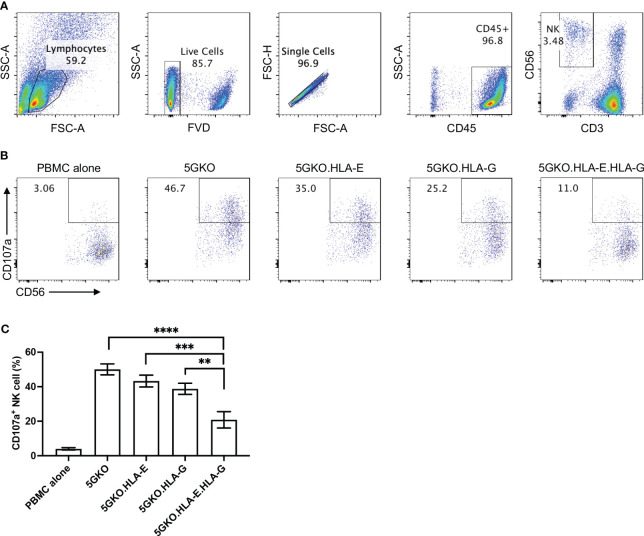
Inhibition of human NK cell degranulation by 5GKO cells expressing HLA-E and HLA-G molecules. rhIL-2 treated PBMCs (n=5) were co-cultured with 5GKO, 5GKO.HLA-E, 5GKO.HLA-G, and 5GKO.HLA-E.HLA-G cells for 2 hours. NK cell degranulation was accessed by CD107a surface expression. **(A)** Gating strategy to identify and refine NK cell population. **(B)** Representative flow plots showing NK cell degranulation in response to the simulation of the modified porcine cells by assessing the percentage of CD107a positive cells in CD3^-^CD56^+^ population. PBMC alone was used as a control. **(C)** Three independent experiments were performed to evaluate NK cell degranulation. Data presented as mean ± SEM. Ordinary one-way ANOVA multiple comparisons test was used to analyze the differences among multiple groups. 5GKO.HLA-E.HLA-G was selected as a control group. **p < 0.01; ***p < 0.001; ****p < 0.0001.

Human NK cell response to pig cell stimulation was examined by CD107a expression on NK cells. Our previous study demonstrated that 5GKO cells, like WT and TKO (triple-gene knockout, *GGTA1/CMAH/β4galNT2*) cells, could activate human NK cell. Despite the elimination of four xenoantigens (aGal, Neu5Gc, Sda, and SLA-I), 5GKO cells maintained the capability to trigger human NK cell degranulation (36). 5GKO cells were used as a control in this study. The gating strategy to identify the NK cell population (CD3^-^CD56^+^) was shown in **Figure 4A**. Representative flow plots showing human NK cell degranulation in response to stimulation by each modified cell line as assessed by the percentage of CD107a positive cells in CD3^-^CD56^+^ population were shown in **Figure 4B**. Ordinary one-way ANOVA multiple comparisons indicated that co-expression of HLA-E and HLA-G on 5GKO cells significantly inhibited CD107a expression on human NK cell compared to 5GKO (p < 0.0001), 5GKO.HLA-E (p < 0.001), and 5GKO.HLA-G (p < 0.01) (**Figure 4C**). Further Student’s t-test indicated that HLA-G expression on 5GKO cells significantly inhibited CD107a expression on NK cells compared to 5GKO cells (p <0.05) while HLA-E expression on 5GKO failed to inhibit CD107a expression on NK cells compared to 5GKO cells (p = 0.1853).

### Human sera antibodies did not react to HLA-E and HLA-G molecules on 5GKO cells

**Figure 5:**
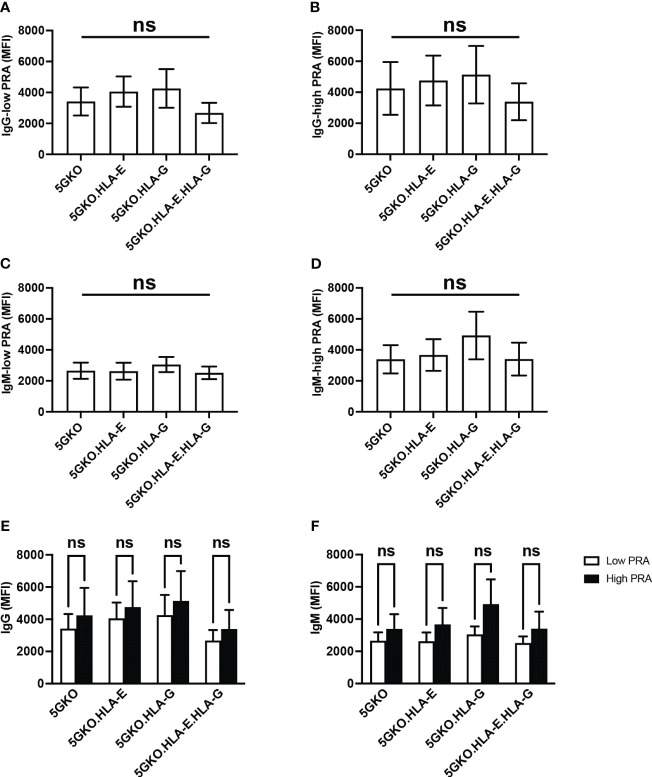
Human antibody reactivity to 5GKO cells expressing HLA-E and/or HLA-G molecules. 5GKO, 5GKO.HLA-E, 5GKO.HLA-G, and 5GKO.HLA-E.HLA-G cells were incubated with heat-inactivated human sera (n = 20), and then stained with goat anti-human IgG Alexa Fluor 488 or donkey anti-human IgM Alexa Fluor 647. Each cell line stained with the secondary antibody was used as a negative control. Human antibody binding was assessed by flow cytometry. Data presented as mean ± SEM. Ordinary one-way ANOVA multiple comparisons test was used to analyze the differences in **(A)** IgG binding with low PRA sera, **(B)** IgG binding with high PRA sera, **(C)** IgM binding with low PRA sera, and **(D)** IgM binding with high PRA sera, among multiple groups. **(E)** Comparison of low PRA sera and high PRA sera in IgG binding to individual modified pig cells was analyzed by the Student’s t-test. **(F)** Comparison of low PRA sera and high PRA sera in IgM binding to individual modified pig cells was analyzed *via* Student’s t-test. ns, not significant.

To test whether non-classical HLA class I molecules HLA-E and HLA-G could react to pre-existing HLA class I antibodies, human antibody (IgG and IgM) binding to 5GKO, 5GKO.HLA-E, 5GKO.HLA-G, and 5GKO.HLA-E.HLA-G cells was examined. A total of twenty human sera samples including ten high PRA sera and ten low PRA sera from the patients on the kidney transplant waitlist were used in this experiment. No statistically significant differences in human IgG or IgM binding among groups were observed: IgG with low PRA sera (p = 0.66), IgG with high PRA sera (p = 0.88), IgM with low PRA sera (p = 0.88), and IgM with high PRA sera (p = 0.75) (**Figures 5A–D**). No statistically significant difference was observed among groups in human IgG or IgM binding with the combination of the high PRA and low PRA sera (data not shown). In addition, there were no significant differences between low PRA sera and high PRA sera in IgG binding to 5GKO (p = 0.65), 5GKO.HLA-E (p = 0.67), 5GKO.HLA-G (p = 0.56), 5GKO.HLA-E.HLA-G (p = 0.53) (**Figure 5E**) as well as IgM binding to 5GKO (p = 0.48), 5GKO.HLA-E (p = 0.37), 5GKO.HLA-G (p = 0.28), 5GKO.HLA-E.HLA-G (p = 0.43) (**Figure 5F**). These results indicate that HLA-E and HLA-G on porcine cells do not react to existing antibodies in human sera, even from highly sensitized individuals.

## Discussion

Expressing inhibitory ligands on porcine cells to induce human NK cell tolerance is a practical approach to protect xenografts from human NK cell-mediated destruction (43). We showed that co-expressing HLA-E and HLA-G on a genetically modified 5GKO cell line synergistically reduced human NK cell activation as compared to cells expressing either HLA-E or HLA-G alone as well as 5GKO parental cells. Our study indicated that HLA-E and HLA-G on porcine endothelial cells did not react to human sera antibodies, and the expression of HLA-E and HLA-G is unlikely to elicit antibody-mediated immune responses.

HLA-E and HLA-G are immunoregulatory molecules and their cooperation has been found in immunosuppressive environments, including physiological (immune tolerance at maternal/fetal interface during pregnancy) and pathological (immune evasion of both tumor and viral infection) conditions (44). The role of HLA-E and HLA-G in inhibiting human NK cell activation has been previously demonstrated in xenotransplantation research (10, 16, 17, 26, 33). In the current study, we found that co-expression of HLA-G and HLA-E on porcine endothelial cells effectively inhibited human NK cell degranulation. HLA-E stability and abundance on porcine cells play a key role in inhibiting human NK cell activation. HLA-E expression level was much higher on 5GKO.HLA-E.HLA-G cells, compared to 5GKO.HLA-E cells. HLA-G VL9 peptides were constantly generated from endogenous HLA-G molecules in 5GKO.HLA-E.HLA-G cells, which could stabilize HLA-E molecules and increase HLA-E expression. A surplus of HLA-G VL9 peptides may be the primary mechanism for the robust increase in cell-surface HLA-E. HLA-E expression on 5GKO.HLA-E cells could also be enhanced by pulsing exogenous HLA-G VL9 peptides in a dose-dependent manner. The stabilized HLA-E-VL9 complexes engage with human NK cell inhibitory receptor NKG2A to protect healthy cells from NK cell-mediated lysis. Previous study indicated that the HLA-E molecules loaded with the HLA-G VL9 peptides exhibited the highest affinity to inhibitory receptor NKG2A compared to the VL9 peptides from HLA-B and HLA-C (32). This magnified inhibition was not associated with HLA-E and HLA-G co-localization on pig cells when co-cultured with human NK cells (unpublished data). In addition, HLA-E and HLA-G interact with different inhibitory receptors on human NK cells through CD94/NKG2-dependent and independent pathways (17, 45). Therefore, co-expression of HLA-E and HLA-G on pig cells leads to a synergistic reduction in human NK cell activation and may provide a novel approach to effectively protect xenografts from human NK cell-mediated cytotoxicity.

In this study, human antibodies (both IgG and IgM) did not react to HLA-E and HLA-G on pig cells. Even across the stratification of human sera with low PRA and high PRA, there was no appreciable increase in antibody binding to HLA-E and HLA-G molecules. These findings suggest that even in highly sensitized individuals, there would likely be no substantial increase in antibody-mediated rejection induced by porcine organs expressing HLA-E and HLA-G.

In human allotransplantation, HLA-E and HLA-G expression can be used to predict transplant outcomes. For example, elevated HLA-G in allografts and in the circulation of recipients was associated with improved graft acceptance in solid organ transplantation (46). In contrast, increased HLA-E expression was found in acute cellular rejection (ACR) biopsies compared to biopsies with no rejection signs, which was correlated with numbers of HLA class I leader peptide mismatches and reduced renal allograft survival (47). Interaction of HLA-E with mismatched HLA class I leader peptides with activating NKG2C receptor may contribute to graft rejection. Recent study demonstrated that mouse and human antibody could bind HLA-E-VL9 complex and enhance NK cell cytotoxicity (48). In xenotransplantation, HLA-E-VL9 complexes could be designed and engineered in pig with the purpose of avoiding antibody binding, facilitating NKG2A interaction, and promoting NK cell inhibition.

The next step will be generating transgenic pigs co-expressing HLA-E and HLA-G. Targeting HLA-E and HLA-G genes to the safe harbor loci in the pig genome can control the copy number of transgene while avoiding undesirable position effects. Studies have shown that the porcine Rosa26 locus and elongation factor 1 alpha (PEF1-alpha) locus are ideal for the integration of transgene for constitutive and ubiquitous expression (49, 50). CRISPR/Cas9-directed targeting of HLA-E and HLA-G constructs to these loci would facilitate transgenic pig production.

In conclusion, our results provide valuable insight into potential mechanisms for overcoming human NK cell-mediated immune rejection in xenotransplantation. Further optimization of this approach, in addition to *in vivo* validation studies, will provide context for the clinical applicability of these GMs in pig-to-human xenotransplantation. The field of xenotransplantation is rapidly progressing, and systematically evaluating potential GMs to optimize pig-to-human compatibility will be crucial to addressing the organ shortage.

## Data availability statement

The datasets presented in this study can be found in online repositories. The names of the repository/repositories and accession number(s) can be found below: https://www.ncbi.nlm.nih.gov/genbank/, NM_001363567.2 https://www.ncbi.nlm.nih.gov/genbank/, NM_004048.4 https://www.ncbi.nlm.nih.gov/genbank/, NM_005516.6.

## Ethics statement

The studies involving human participants were reviewed and approved by the Institutional Review Board (IRB) of Indiana University (IRB#11013).

## Author contributions

AC-N, KF, AI, JW, and PL performed experiments, AC-N, WL, WZ, KL, YP, NH, and PL analyzed data. AC-N, WL, ZL, and PL interpreted data. AC-N and PL wrote the paper. DC provided guidance and critical review of the manuscript. PL and BE conceived of the study and provided funding. PL designed experiments and supervised the project. All authors contributed to the article and approved the submitted version.
